# Fondaparinux–Associated Rectus Sheath Hematoma: Skating on Thin Ice

**DOI:** 10.7759/cureus.7938

**Published:** 2020-05-02

**Authors:** Nikolaos Sabanis, Aikaterini Drylli, Vasiliki Mamakou, Eleni Paschou, Georgios Zagkotsis

**Affiliations:** 1 Nephrology, General Hospital of Livadeia, Livadeia, GRC; 2 Otorhinolaryngology, National and Kapodistrian University of Athens, Athens, GRC; 3 Internal Medicine, Dromokaiteio Psychiatric Hospital, Athens, GRC; 4 Family Medicine, Medical Unit of Saint George, Livadeia, GRC

**Keywords:** rectus sheath hematoma, fondaparinux, acute kidney injury

## Abstract

Rectus sheath hematoma (RSH), an exceptionally rare clinical entity, results from the rupture of epigastric arteries or tear of the rectus abdominis muscle itself. Spontaneous RSH represents a potentially life-threatening bleeding complication in anticoagulated patients with distinct characteristics. The non-specific nature of RSH clinical manifestations renders RSH a kaleidoscopic disease that may be misdiagnosed. The widespread use of anticoagulants for deep vein thrombosis (DVT) prophylaxis or therapy is among the most commonly documented risk factors. To the best of our knowledge, this is the first report of a fondaparinux-associated giant RSH in a 58-year-old Caucasian man who presented with severe pain at the right abdominal quadrant accompanied with a large ecchymosis secondary to violent cough due to a respiratory infection. The aim of our study is to broaden current knowledge regarding the predisposing factors, the pathophysiological mechanisms, and the management of this bleeding disorder.

## Introduction

Rectus sheath hematoma is considered a rare clinical entity and an intriguing diagnostic dilemma, despite the fact that until now, a remarkable number of case reports, case series, and retrospective observational studies have been witnessed. RSH is originated from the bleeding into the rectus sheath after damage to the superior epigastric artery and inferior epigastric artery or their rich anastomoses near the level of the umbilicus. Apart from external trauma and iatrogenic causes, a plethora of non-traumatic RSH cases have also been discussed in the literature [1].

Anticoagulant-associated RSH tends to become an increasingly recognized hemorrhagic complication of anticoagulation treatment for deep vein thrombosis (DVT) prophylaxis or therapy. Vitamin Κ antagonists, low molecular weight heparins (LMWHs), thrombin inhibitors and other oral factor Xa inhibitors have been reported to cause RSHs in anticoagulated patients. The risk of developing RSH is multifactorial and involves several predisposing factors that are orchestrated by the intensity and type of anticoagulant [2]. Advanced age, female gender, chronic kidney disease, immunosuppressant or steroid therapy as well as inappropriate subcutaneous abdominal injection of heparins or concomitant use of antiplatelet agents constitute well-known precipitating factors of this uncommon bleeding complication [3,4].

Fondaparinux is a selective indirect inhibitor of factor Xa, and its action occurs after binding to the pentasaccharide binding site of antithrombin-III (AT-III). Thereafter, fondaparinux inhibits thrombin generation and does not affect thrombin activity. That remarkable feature of fondaparinux explains its widespread use in clinical practice for the treatment and prevention of DVT and pulmonary embolism, without significant risk of major bleeding consequences in contrast to LMWHs [5]. With this in mind, we believe that we have found an exceptional case of fondaparinux-associated giant bilobar RSH. To the best of our knowledge, this is the first report that highlights the need for surveillance and high clinical suspicion of RSH in vulnerable patients on any anticoagulant therapy.

## Case presentation

**Figure 1:**
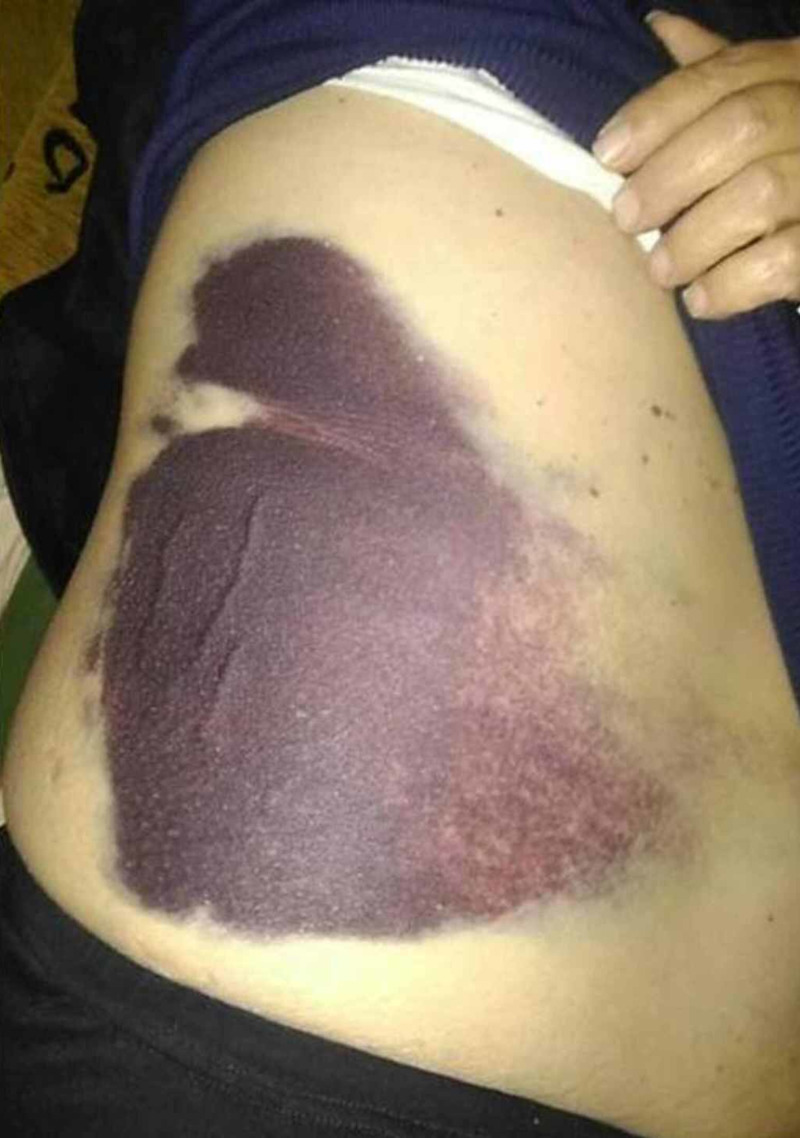
Rectus sheath hematoma (RSH): ecchymosis of the right abdominal wall

A 58-year-old, obese, Caucasian man presented to the emergency department, complaining of severe abdominal pain associated with generalized weakness and occasional vomiting. Abdominal pain started quite suddenly, two days before his admission, and had increased gradually since then. The pain was non-radiating or associated with food intake, whereas its location witnessed to be mainly at the right lower abdominal quadrant. Notably, it was accompanied with a feeling of distension. Simultaneously, an extremely large ecchymosis (Figure 1), extended from the right lower anterior abdominal wall to the external abdominal oblique muscles unilaterally, was observed.

The patient reported that two weeks previously had an upper-respiratory-tract infection, which lasted about five days and consisted of a sore throat, sneezing, and violent coughing spells. He also had a tendency to be constipated, and this had become more of a problem recently.

His medical history included a ten-year history of resistant hypertension due to primary hyperaldosteronism and chronic kidney disease stage III with recent creatinine levels at 1.7 mg/dL. An acute ischemic stroke three years prior to his admission was also reported, without significant residual neurological deficits. Additionally, he reported a two-year history of type II diabetes mellitus and a long-lasting history of chronic obstructive pulmonary disease. Despite that, the patient smoked 10-20 cigarettes per day for 35 years and drank alcohol occasionally. No family history of inherited bleeding disorders or a relevant thrombophilic tendency was recorded. 

Furthermore, five months ago, the patient had been hospitalized for deep vein thrombosis and an associated asymptomatic episode of pulmonary embolism, which was investigated thoroughly in order to exclude any secondary cause of thromboembolism. Afterward, the patient was discharged and treated with fondaparinux at a daily dose of 10 mg subcutaneously. It is noteworthy that no dose adjustment was adhered regarding his estimated glomerular filtration rate (eGFR) (44.2 ml/min/1.73m^2^). In terms of his long term medication, the patient was treated on a regular basis with spironolactone, furosemide, amiloride, amlodipine, esomeprazole, nebivolol, low dose of aspirin, atorvastatin and alogliptin as well as salmeterol and fluticasone inhalation.

On physical examination, the patient looked unwell, pale, sweaty, and in pain. His temperature was 37.8°C and his pulse was low-volume, regular rhythm and rate was 104 bpm despite the fact that he was on oral β-blocker therapy. Blood pressure was 92/68 mmHg lying and 65/50 mmHg sitting up. His jugular venous pressure was not raised, and heart sounds were normal. On chest auscultation, he had expiratory polyphonic wheezes, but no crackles, and his respiratory rate was 28/min with SpO2 95%. His abdomen was distended with guarding and rebound tenderness, whereas a highly painful bulging area was noted in the lower abdominal quadrants extending bilaterally. A positive Carnett’s sign was also observed, and faint bowel sounds were audible.

Routine laboratory tests revealed normochromic, normocytic anemia with a hematocrit of 20.8%, decreased hemoglobin of 7.1 g/dL, a platelet count of 181 x 10^3^/μL and high levels of inflammation markers (white cell count of 18.88 x 10^3^/μL, erythrocyte sedimentation rate of 62 mm/h, and C-reactive protein of 3.5 mg/dL). International normalized ratio (INR) and activated partial thromboplastin time were 1.09 and 28 sec, respectively, whereas the fibrinogen levels were also within normal values. At admission, kidney function was significantly impaired since creatinine levels had been raised up to 3.9 mg/dL and blood urea levels up to 180 mg/dL. Liver-function tests did not reveal evidence of dysfunction, and urinalysis was compatible with pre-renal acute kidney injury since an elevated urine osmolarity (urine specific gravity of 1.030) and a decreased urinary excretion of sodium (6 mEq/L) were observed. No electrolyte abnormalities were recorded despite the fact that the patient on admission was oliguric with acute kidney failure and received two different potassium-sparing diuretics. Mild hyperchloremic metabolic acidosis (pH: 7.33) was noted, accompanied with decreased serum bicarbonate (HCO3: 9.9 mmol/L) and pCO2 (16.3 mmHg) levels in addition to moderately increased lactate levels (7.4 mmol/L). Therefore, the calculated increase of anion gap that was recorded could undoubtedly be ascribed both to acute renal failure and hypovolemic shock.

**Figure 2:**
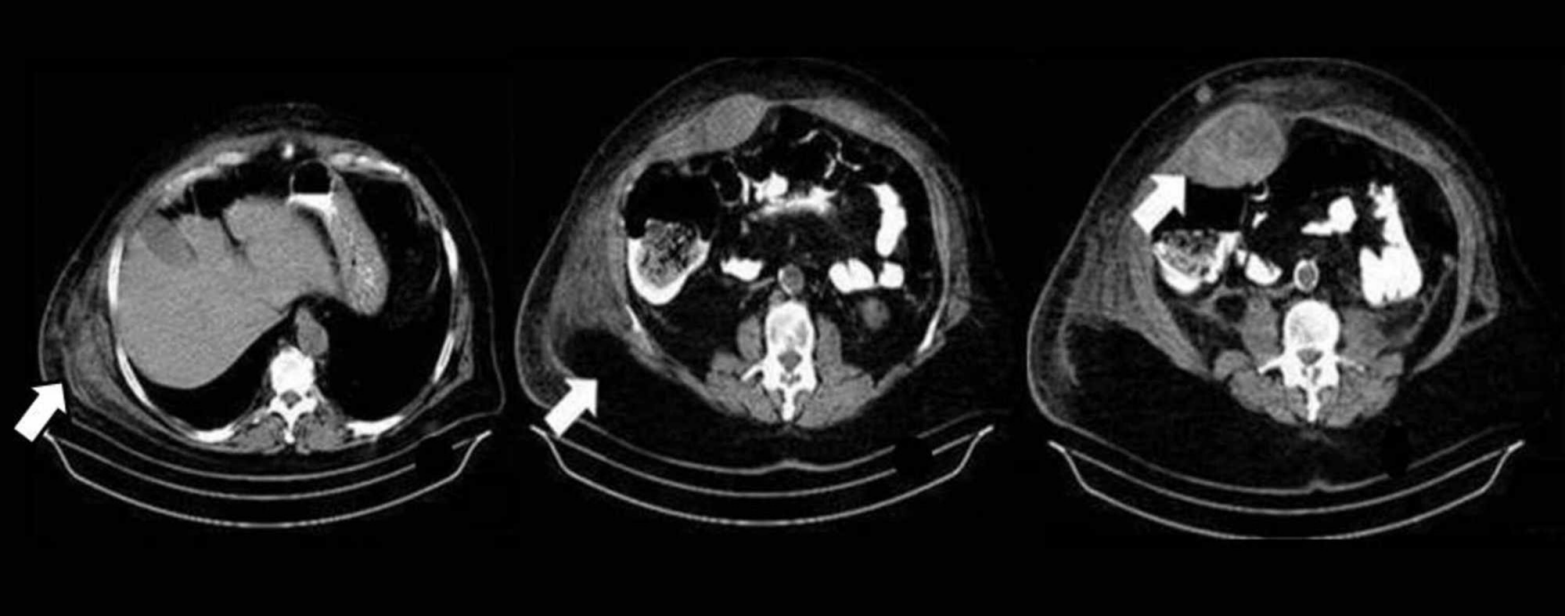
Computed tomography: giant bilobar RSH extended to internal and external oblique muscles, right transversus abdominis muscle, and subcutaneous fat tissue

Afterward, both abdominal ultrasonography and abdominopelvic computed tomography (CT) scan were pursued in order to rule out any intra-abdominal cause of acute abdominal pain. Even though abdominal ultrasonography was inconclusive, CT scan revealed a giant rectus sheath hematoma measuring 32x10x19 cm with a fluid-fluid level. Remarkably, the CT scan showed RSH outspreading bilaterally from the right-sided internal and external oblique muscles to the right transversus abdominis muscle and subcutaneous fat tissues (Figure 2).

Certainly, the diagnostic value of CT scan was limited, as it was performed without intravenous contrast due to underlying renal insufficiency. Besides that, there was no evidence of intraperitoneal bleeding.

Provided that a confirmed diagnosis had been made, an immediate conservative therapy had been also considered to restore the intravascular volume. Isotonic crystalloid solutions infused rapidly based on regular monitoring of systolic blood pressure, urine output, and pulse pressure fluctuation. Due to hyperchloremic metabolic acidosis, we infused not only normal saline solutions but also lactated Ringer’s solutions. In addition, the patient received overall four packed red blood cell units and four fresh frozen plasma units during the first two days of hospitalization, based on a balanced transfusion pattern 1:1. In this way, we managed to avoid further metabolic abnormalities or even hemodilution and resuscitation-induced coagulopathy. Indeed, no hemostatic derangements were observed during hospitalization, and eventually, the patient’s hemodynamic stability and renal function recovered gradually. On the sixth day of hospitalization, the patient was discharged home with a hemoglobin level of 10.2 g/dL and remained stable on regular follow-up one week later (hemoglobin of 10.4 g/dL).

As far as anticoagulation therapy was concerned, we felt like skating on thin ice, given that our patient had a recent history of deep vein thrombosis associated with an asymptomatic episode of pulmonary embolism, as well as a previous acute ischemic stroke. Once fondaparinux and low dose of aspirin were stopped, the patient was at a high risk of thromboembolism. Thereafter, a jeopardized therapeutic decision was made at discharge, and the patient received apixaban at a dose of 2.5 mg twice a day. At one month follow-up, the patient remained symptomless despite that a moderate, painless mass was still evident at the right lower abdominal quadrant. 

## Discussion

RSH is a rare and potentially devastating bleeding complication, defined as the accumulation of blood into the rectus sheath after disruption of the superior epigastric artery and inferior epigastric artery or their rich anastomoses near the level of the umbilicus. Apart from meta-traumatic and iatrogenic causes, there is an unambiguous relationship between RSH and various chronic medical conditions [1].

The etiopathogenesis of RSH involves a large number of co-existing predisposing factors that interact and incite the rupture of an epigastric artery’s branch. Inferior epigastric artery’s branches consist, anatomically, the most vulnerable vessels to shear forces, and for this reason, the most common site of RSH formation seems to be the lower abdomen quadrant. Under these conditions and once a branch is ruptured, bleeding occurs, and in case of inappropriate compression of bleeding, the hematoma is expanded and becomes symptomatic [1,6].

The RSH formation and expansion have been correlated to several predisposing factors that are usually interrelated and synergistically incite, on one side, the disruption of an epigastric artery’s branch and, on the other side, the imbalance of coagulation cascade [7]. Previous studies have reported a significant number of RSH-associated risk factors that generally are divided into two categories, traumatic and non-traumatic ones. Non-traumatic causes of RSH involve various underlying medical conditions such as anticoagulation therapy or infection-related coagulation abnormalities [8,9].

However, the most frequently implicated medical condition is bouts of severe coughing during the flares of asthmatic bronchitis or upper respiratory tract infections [10]. Similarly, strenuous and uncoordinated rectus abdominis muscle contractions that occur during straining from constipation or sports activities have also been considered to provoke RSH formation [11]. As described in our patient, an exacerbation of chronic obstructive pulmonary disease after an upper respiratory tract infection was observed concomitantly with a period of severe straining from constipation.

Although RSH pathogenesis is multifactorial, the fundamental role of anticoagulation on the increasing prevalence of RSH in anticoagulated patients is also well-documented. This is exemplified in the work undertaken by Sheth and colleagues, wherein more than three-fourth (77.4%) of the patients were on anticoagulation therapy [12]. Similarly, the close relationship between anticoagulation and RSH has been widely investigated by Hatjipetrou and colleagues who reviewed 146 articles and identified the use of anticoagulation therapy as an indisputable predisposing factor for RSH and probably the most common one [6]. As pointed out in the same review, in RSH patients under anticoagulation therapy, hemorrhage volume is large, resulting in increased morbidity and mortality.

Anticoagulant-related RSH cases include almost all different types of anticoagulants: Vitamin K antagonists, low molecular weight heparins or unfractionated heparins, and novel direct oral thrombin inhibitors or factor Xa inhibitors [7,13]. Herein, as the use of various anticoagulant agents rises rapidly world-wide, one may consider that the incidence of RSH also increases. Indeed, a plethora of anticoagulant-associated RSH cases have been published until now. In the same context, it is also well-documented that distinct predisposing factors are involved in anticoagulant-associated RSH formation: advanced age, abdominal obesity, use of concurrent medications and especially antiplatelet agents, specific comorbid diseases such as hypertension and arteriosclerosis, and of course chronic kidney disease with estimated glomerular filtration rate less than (30 ml/min/1.73m^2^) [6]. The evidence presented in our case thus far supports the concept that RSH development requires various predisposing factors and precipitating events that act simultaneously on a susceptible person.

As far as fondaparinux-associated RSH is concerned, it is well known that fondaparinux represents a selective indirect factor Xa inhibitor that shares all biologic and pharmacologic advantages of LMWHs without inhibition of thrombin or antigenic properties. Thus, fondaparinux anti-Xa activity is approximately seven-fold higher than that of LMWHs, and heparin-induced thrombocytopenia (HIT) seems to be negligible when fondaparinux is used for the therapy or prevention of venous thromboembolism [14]. Nowadays, fondaparinux is used more frequently in stable patients, whereas the main contraindication for its use is severe kidney impairment since fondaparinux is excreted unchanged in the urine. In the case of moderate kidney impairment (30-50 ml/min), the dose of fondaparinux should be reduced by half in order to avoid the risk of a major bleeding episode [5].

It could be hypothesized that in our patient, the RSH formation was mainly the result of a non-strict dose adjustment of fondaparinux to his baseline eGFR. Given that, the expansion of hematoma deteriorated rapidly, causing hemodynamic instability and a significant reduction in renal function. Once the glomerular filtration rate aggravated, the anti-Xa activity of fondaparinux increased. Certainly, in our case, RSH expansion could also be attributed to platelet dysfunction due to the concomitant use of aspirin and the uremic milieu of acute renal failure.

From a clinical point of view, symptomatic RSH is usually a self-limiting bleeding complication that tamponaded efficiently within rectus sheath and less commonly follows a life-threatening clinical course due to rampant expansion [15]. Infrequently, RSH may cross the midline and become bilateral, as occurs in our case description.

Regarding the clinical picture, the majority of RSH patients complain of acute abdominal pain and an associated palpable abdominal mass, whereas, less frequently, they report other constitutional, gastrointestinal, or even urologic symptoms attributable to the severity of bleeding and the degree of peritoneum irritation. The nonspecific nature of RSH clinical manifestations is responsible for its description as a kaleidoscopic disease that mimics several acute intra-abdominal pathologies [8]. In these cases, physical exam findings can help distinguish RSH from other common acute intra-abdominal diseases, as occurred in our patient who presented with positive Carnett’s sign. Eventually, the diagnosis of RSH is confirmed by abdominal ultrasonography or computed tomography (CT) scan or both [2]. In our case, abdominal ultrasonography was inconclusive, whereas CT scan revealed a giant bilateral RSH. Indeed, CT scan is the gold standard diagnostic approach of RSH with a sensitivity of up to 100%, while abdominal ultrasonography is considered a diagnostic tool of limited value with a reported sensitivity of approximately up to 70% [16-18].

Whether RSH diagnosis is confirmed, the patient’s clinical status should determine principally what therapeutic decision will be made, either conservative or invasive, through arterial embolization or even open surgical intervention. Recent evidence suggests that conservative care is the choice of treatment of spontaneous abdominal wall hematoma due to anticoagulant/antiplatelet use, whereas an invasive intervention should always be kept in mind for hemodynamically unstable patients [19]. Contrella and colleagues have also evaluated the radiographic, laboratory, and clinical factors associated with conservative management failure in a retrospective study of 72 patients with spontaneous RSH. Notably, the investigators created a scoring system of high sensitivity and specificity to identify RSH patients who would require arterial embolization [20].

In our case, a conservative therapeutic approach was performed, taking into account, on the one hand, the relatively immediate hemodynamic stabilization of the patient and, on the other hand, the rapid improvement of acute kidney impairment at the baseline levels. Therefore, we can hypothesize that rapid kidney function improvement enhanced the clearance of fondaparinux and, as a result, its anticoagulant activity. Besides bleeding, our patient was also a therapeutic challenge since routine tests of coagulation were considered unsuitable, anti-factor Xa assays were unavailable, and chiefly, there was no antidote for this drug.

## Conclusions

RSH is considered the synergistic result of several predisposing factors that follow an unfortunate sequence on a vulnerable anticoagulated patient, and it should be included in the differential diagnostic approach of acute abdominal pain.
